# Dilute-and-Shoot HPLC-UV Method for Determination of Urinary Creatinine as a Normalization Tool in Mycotoxin Biomonitoring in Pigs

**DOI:** 10.3390/molecules25102445

**Published:** 2020-05-24

**Authors:** Agnieszka Tkaczyk, Piotr Jedziniak

**Affiliations:** Department of Pharmacology and Toxicology, National Veterinary Research Institute, 24-100 Pulawy, Poland; piotr.jedziniak@piwet.pulawy.pl

**Keywords:** pig urinary creatinine, HPLC-UV method, mycotoxin biomonitoring, urine standardization

## Abstract

A simple, rapid, and accurate HPLC-UV method was developed for the determination of creatinine in pig urine. Usually, it is determined in urine in biomonitoring of xenobiotics to correct for variations in dilutions of urine samples. The colorimetric method (based on Jaffe reaction), which was mainly used for this purpose in mycotoxin biomonitoring, is not a reliable approach for pig urine. Therefore, a novel and accurate HPLC method for creatinine determination was developed. The sample preparation was based on the dilute and shoot approach. An HPLC separation was performed with a porous graphitic carbon column with an aqueous mobile phase to achieve satisfactory retention time for creatinine. The method has been successfully validated, applied for the determination of creatinine in pig urine, and compared with other methods commonly used for that purpose—a colorimetric method based on Jaffe reaction and commercial ELISA test. The developed HPLC method shows the highest precision and accuracy for pig urine samples. Finally, the method was applied as a normalization tool in LC-MS/MS mycotoxin biomarkers analysis. The standardization to a constant creatinine level (0.5 mg/mL) enables similar matrix effects for eleven mycotoxin biomarkers for pig urine samples with different creatinine levels.

## 1. Introduction

Creatinine (2-amino-1-methyl-2-imidazoline-4-one) is the final metabolism product of creatine in mammals [1], which is excreted exclusively by the kidneys via glomerular filtration and, to a lesser extent, by tubular secretion [2]. Under physiological conditions, its excretion throughout the day is relatively constant, the amount of creatinine produced is proportional to the muscle mass of the individuals [3]. In contrast, urine production depends on the intake or loss of fluid. Therefore, creatinine concentration in urine can be taken as a measure of the dilution of the urine [4]. The physiological range of creatinine content can differ among the species, from lower values in humans (0.28–2.59 mg/mL) [5] and higher for pigs (0.07–10.77 mg/mL) (our data).

Creatinine concentration is commonly used as a standardization tool for quantifying xenobiotics in urine (biomarkers) [6]. The World Health Organization (WHO) recommends if a sample is too diluted (creatinine concentration < 0.3 mg/mL) or too concentrated (creatinine concentration > 3.0 mg/mL), another urine void should be collected (WHO, 1996). In biomonitoring, spot samples of urine are usually analyzed, since it is not practical to obtain urine samples over long periods [7]. Therefore, dividing biomarker concentration by the creatinine level measured in the same sample can account for variability due to urinary dilution [8]. Thus, an accurate and reliable method for creatinine determination is needed.

Other parameters frequently used to adjust analyte concentrations in urine samples include specific gravity, conductivity, and osmolarity [7,9,10]. Choosing from the above parameters as measures of dilution remains unclear and may depend on the renal elimination mechanism(s) of the biomarker(s) of interest [11,12]. Creatinine adjustment may be used to correct urine concentration results when the substance is eliminated primarily through renal filtration [11,13]. Therefore, analyte concentrations in urine are mainly reported as creatinine-corrected concentrations [14].

Different methods for creatinine determination are available. Spectroscopic methods based on Jaffé’s reaction, where a colored complex of creatinine with picric acid in basic solution is formed and its absorbance is measured at 505 nm wavelength [15]. However, colorimetric methods can be adversely affected by numerous metabolites and drugs found in biological samples [16]. Enzymatic assays like ELISA (enzyme-linked immunosorbent assay), which are commercially available for urine analysis in different species are time-consuming and relatively expensive [17,18].

Alternative approaches include sensitive or selective separation methods such as liquid chromatography (LC) and capillary electrophoresis [6,19]. Recently, sensors and biosensors based on immobilized enzymes or molecularly imprinted polymers (MIPs) were used to analyze creatinine in clinical laboratories [20,21].

Reversed-phase high-performance liquid chromatography (RP-HPLC) [22] and liquid chromatography-tandem mass spectrometry (LC-MS/MS) [23,24,25] have been recently the most popular techniques for the measurement of creatinine in biological samples. They could be alternative techniques for creatinine determination with adequate sensibility and specificity. Recent studies have shown the advantages of hydrophilic interaction chromatography (HILIC) over the RP-HPLC for the separation of highly polar compounds, particularly small biological metabolites [26,27].

In the case of mycotoxin urinary biomarkers in pigs, there are only three studies, in which standardization for different dilutions of urine samples was used. Clinical-chemistry automatic analyzer **[27]**, LC-MS method [28], and the enzymatic method [29] were used for creatinine determination. In human biomonitoring studies, standardization to creatinine level was far more often (Table S1). Frequently, creatinine is determined based on the Jaffe reaction [30,31,32,33,34,35,36]. In some studies, the levels of urinary creatinine were also determined based on a fast LC-MS/MS approach [37,38,39,40] and enzymatic test [41].

In this study, an accurate, robust, and simple LC-UV method based on chromatographic separation with porous graphitic carbon stationary phase was developed for the determination of creatinine in pig urine. The levels of the creatinine in pig urine samples were determined with a developed method and the results were compared with creatinine measurement with two other methods: a spectroscopic method based on Jaffe’s reaction and ELISA test. Additionally, this developed LC-UV method was applied in the analysis of pig urine samples and as a normalization tool in LC-MS/MS mycotoxin biomarkers analysis. The standardization to constant and low creatinine level (0.5 mg/mL) enabled achievement of similar matrix effects for determination of mycotoxins in urine samples with different creatinine levels.

## 2. Results and Discussion

### 2.1. Optimization of Chromatographic Separation

**Figure 1:**
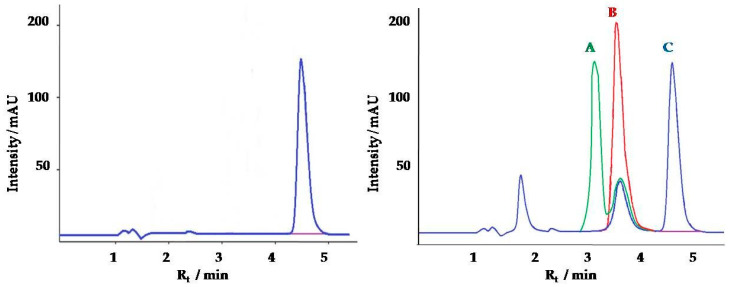
(**a**) Chromatogram of creatinine standard (10 µg/mL, 0.1% TFA, R_t_ = 4.6 min); (**b**) Chromatograms of creatinine extracted from pig urine samples with different TFA content in mobile phase: A—0.01% TFA, R_t_ = 3.1 min, B—0.05% TFA, R_t_ = 3.6 min, C—0.1% TFA, R_t_=4.6 min.

Creatinine is a very polar small molecule and has poor retention on a reversed-phase stationary phase [42]. An alternative to commonly used in reversed-phase chromatography alkyl-bonded (e.g., C18) silicas sorbents is porous graphitic carbon. The method was based on Thermo Scientific Application Note 20512, which was modified and applied for pig urine samples [43]. The mobile phase contains acetonitrile (ACN) and high percentages of water (97%) with the addition of trifluoroacetic acid (TFA) enabled achievement of sufficient retention and peak shape of creatinine on the Hypercarb column. The percentage of TFA—very strong acid—plays a significant role in the retention of creatinine on the Hypercarb column. Ammonium acetate and acids, like formic acid, acetic acid, and TFA are frequently added to mobile phase, to enhance analyte retention and also to improve the peak shape and consequently the chromatographic separation efficiency by protonation of the carboxyl groups [44]. After optimization, 0.1% addition of TFA was optimal to achieve excellent creatinine retention and its separation from matrix components (Figure 1). Obtained retention time (Rt) was 4.6 min—much longer compared to C18 based stationary phase (Rt = 0.5 min) [24] and similar to the HILIC HPLC method (Rt =4 min) [25].

### 2.2. Validation of Chromatographic Method

Good linear regression of the calibration curve was observed - the correlation coefficients were above 0.9988. A typical calibration curve followed the equation:

(1) y=183.3x+28.37.

The limit of detection (LOD) and limit of quantification (LOQ) was 0.3 and 1 µg/mL, respectively. No carry-over effects were detected. The percentage recovery was found to be 89.4–114% for creatinine and day-to-day precision (RSD) based on the retention time and peak area was better than 12.0% (Table 1). These values are similar to those obtained with the HILIC LC-UV method [25]. LOQ obtained with Disposable Pipette Extraction HPLC method is much higher (0.3 mg/mL) than this developed method [22].

### 2.3. Comparison of the Creatinine Concentration Results in Pig Urine Samples Obtained with LC-UV, Spectroscopic and ELISA Method

**Figure 2:**
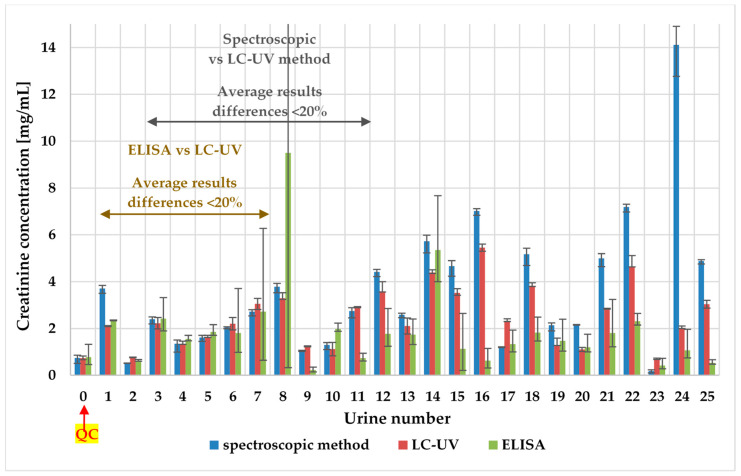
The pig urinary creatinine levels—comparison of spectroscopic, ELISA, and LC-UV method. For every method, the scatter of the results is shown.

The concentrations of creatinine in tested pig urine samples with spectroscopic, LC-UV, and ELISA methods are given in Figure 2.

ELISA assay dedicated to pig urine samples couldn’t be reproducible in an analysis of real pig urine samples—RSD lower than 20% was only for 35% of samples (9/26). Although, in each batch, biochemistry control urine (QC, urine number 0 in Figure 2) was added, and its concentration was consistent with producer data. The explanation for it could be the complexity of matrix—pig urine samples and possible cross-reactivity between antibodies and similar structure compounds [45].

We can observe that LC-UV (RSD = 0.6–13.8%), as well as the spectroscopic method (RSD = 0.5–13.7%), provide repeatable results (RSD < 15%) for all analyzed samples in contrast to the ELISA test (RSD = 1.6–84%). Results obtained with the spectroscopic method are consisted (differences lower than 20%) with the LC-UV method only for 38.5% of the samples (10/26). Results obtained with ELISA test consist of an LC-UV method (differences lower than 20%) for 80% of the samples (8/10), which were replicated (only 38.5% of analyzed samples—10/26). For the biochemistry control urine, all methods perform well (RSD < 20%).

Although for biochemistry control urine spectroscopic as well as LC-UV method performs well. There are huge differences in results between those methods in the analysis of real urine samples. The reason for this fact could be, that the Biosystems kit is recommended for human urine and our results confirm that these kits are not suitable for pigs’ urine. Creatinine levels in pig urine samples are much higher than in human urine [46] and another validation for pig urine samples is needed. Additionally, various endogenous and exogenous compounds interfere with these photometrical measurements in biological fluid samples [27,47]. The problem with Jaffe-based creatinine methods was also observed in human serum in terms of imprecision and lack of specificity—this variability brings into question their clinical utility. It is recommended to replace Jaffe-based creatinine methods with more specific methods that are enzyme-based [48].

The developed LC-UV method was chosen for future analysis and successfully validated for pig urine samples.

### 2.4. Application of the LC-UV Method to the Pig Urine Samples

**Figure 3:**
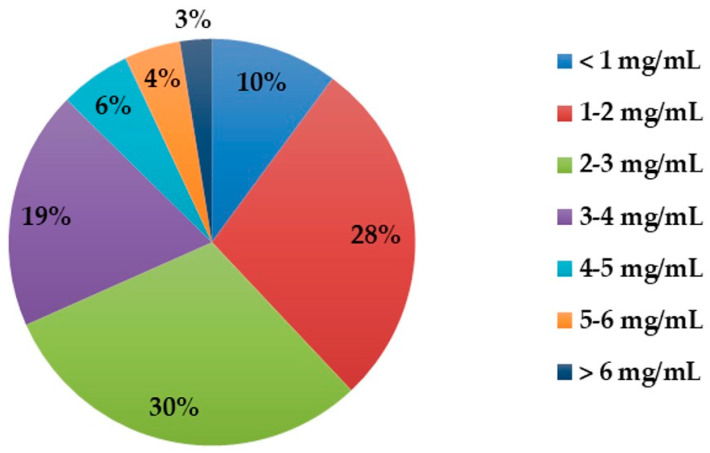
Levels of creatinine in 166 pig urine samples, determined with the LC-UV method.

The developed LC-UV method was applied to the analysis of creatinine in 166 pig urine samples. The concentrations of creatinine in tested pig urine samples are given in Figure 3.

The concentrations of creatinine measured were in the range of 0.07 to 10.77 mg/mL. The values were consistent with the ranges reported in the literature [49] and much higher than the physiological range for urine creatinine is 2.5–23 mM (0.28–2.59 mg/mL) in human urine [5]. This difference can be the reason the spectroscopic method and ELISA failed with pigs’ urine.

### 2.5. Standardization of Urine Samples to the Constant Creatinine Level for LC-MS/MS Analysis of Mycotoxin Biomarkers

Biomonitoring data usually are adjusted to a constant creatinine concentration to correct for variable dilutions among samples. The ratio between toxin concentration and creatinine content of urine is the most popular way to correct for the different water content of human and animal urine in biomonitoring of mycotoxins.

As shown in Table S1, in human urine, creatinine levels are rather low and constant—in the range: 0.8-1.7 mg/mL (mainly based on the Jaffe reaction). Therefore, only a slight improvement could be achieved by taking the creatinine concentration into account. The same situation was observed in the case of bovine urine [50]. The matrix effect was reported in only one of the examination studies.

One of the main challenges in LC-MS/MS multi-mycotoxin method development was a high diversity of validation results for different pig urine samples in terms of matrix effects.

Up to now, there are only two studies, in which pig urine samples were diluted to the same creatinine content (determined by Jaffe and LC-MS/MS method) with water to obtain similar concentrations of matrix compounds in all samples. Urine was diluted before analysis to a creatinine content of 0.2 mM [28,29].

**Figure 4:**
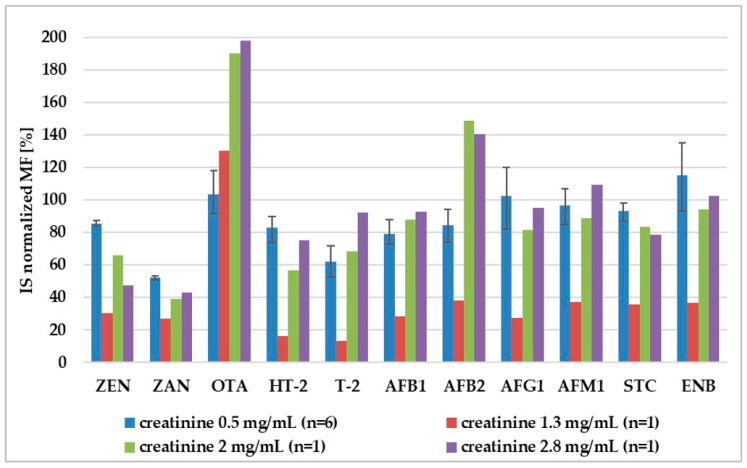
Comparison of internal standard-normalized matrix factor (IS-normalized MF) for normalized (urinary creatinine = 0.5 mg/mL) and non-diluted urine (urinary creatinine = 1.3, 2, 2.8 mg/mL). For creatinine 0.5 mg/mL—the scatter of the results is shown, because IS-normalized matrix factor was calculated for six different urine samples. Adjustment to other creatinine levels (1.3, 2, and 2.8 mg/mL) was tested only on one urine sample.

**Figure 5:**
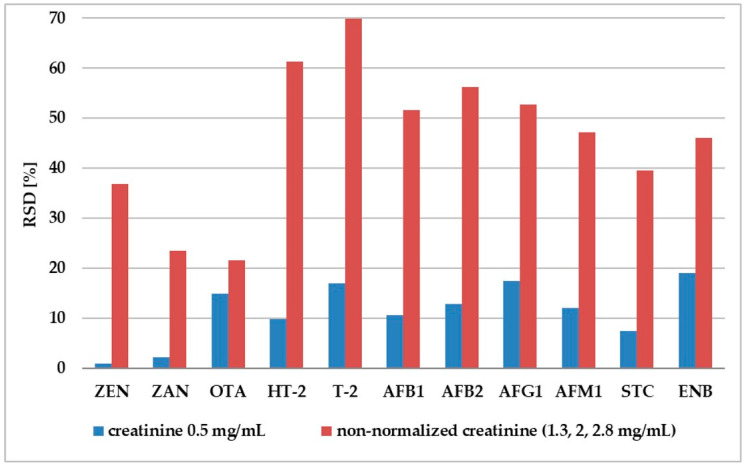
Comparison of day-to-day precision (RSD) values of matrix factor for normalized (urinary creatinine = 0.5 mg mL^−1^ in six replicates) and non-normalized urine (variance between results of mycotoxin analysis in three different creatinine levels: 1.3, 2, 2.8 mg mL^−1^).

Our experiment shows that adjustment to 0.5 mg/mL of creatinine enabled standardization of internal standard normalized matrix factor (IS-normalized MF) for different pig urine samples (Figure 4). For all analytes, the RSD of the IS-normalized MF calculated from the six lots of samples was lower than 20%, which enabled meeting the EMEA criteria in terms of matrix effect. For non-diluted urine, the differences in IS-normalized MF for urine with different creatinine levels (1.3, 2, 2.8 mg/mL) were much higher than 20%. (Figure 5).

## 3. Materials and Methods

### 3.1. Chemicals

The creatinine reference compound was obtained from Sigma Aldrich. HPLC-grade acetonitrile was purchased from J.T. Baker (Phillipsburg, NJ, USA). Trifluoroacetic acid was obtained from Fluka (Buchs, Switzerland). All other chemicals were of analytical grade. The water was purified using a Direct-Q water purification system (Millipore, Bedford, MA, USA).

Reagents for measurement of creatinine concentration (COD 11802, COD 11502, COD 11542) and biochemistry control urine (COD 18054) were obtained from BioSystems S.A. (Barcelona, Spain) and stored at 2–8 °C.

Porcine creatinine ELISA kit was provided from SunLong Biotech Co., Ltd. (Hangzhou, China) (SL0182Po). It contained micro ELISA strip plate, standard (180 nmol/mL), standard diluent, sample diluent, and other reagents used in creatinine assay.

### 3.2. Preparation of Standard Solutions for Chromatographic Method

A stock solution of creatinine was prepared at a concentration of 1 mg/mL in deionized water. The working solutions of various concentrations (100, 50, 10, 5, 1 µg/mL) of creatinine were prepared by the appropriate dilution with deionized water. All of these solutions were stored at 4–8 °C in the refrigerator for three months.

### 3.3. Urine Samples

Urine samples (*n* = 166) collected from pigs before slaughtering between January and September 2019. The samples were taken by Veterinary Inspection in Poland under the Residue Control Monitoring Program. The samples were stored in the refrigerator at −20 °C until analysis.

### 3.4. Sample Preparation

#### 3.4.1. Chromatographic Method

Urine samples were centrifuged to obtain clear supernatants at 4500*g* for 15 min. Then, 10 μL of urine was diluted with 990 µL of mobile phase (Section 3.5). The aliquots of diluted urine samples were centrifuged at 14000 rpm for 10 min and transferred to autosampler vials. An aliquot (25 μL) of the sample solution was injected into the LC-UV for analysis.

#### 3.4.2. The Spectroscopic Method Based on Jaffe Reaction

Urine samples and working reagents were prepared according to the Biosystem (Spain) procedure. Urine samples were centrifuged for 15 min at 4500 rpm and diluted 1:200 with distilled water. Biochemistry control urine was diluted 1:50 with distilled water. The working reagent and the photometer were brought to 37 °C. Then, 1.0 mL of working reagent and 0.1 mL of standard/diluted urine sample were mixed and pipetted into a cuvette.

#### 3.4.3. ELISA Test

Pig urine samples were centrifuged for 15 min at 4500 rpm and diluted 1:500 with distilled water. A five-point calibration curve (0.001–0.014 mg/mL) and pig urine samples were prepared in three replicates according to the procedure [51]. In each batch, the absorbance of biochemistry control urine was measured.

### 3.5. Chromatographic Conditions

The Agilent 1200 HPLC system (Agilent Technologies, Waldbronn, Germany) consisted of a handheld control module, quaternary gradient pump, a vacuum degasser, an autosampler, thermostated column compartment, and UV detector.

The analytical column was a Hypercarb column (100 × 2.1 mm, 5 μm, Thermo Fisher Scientific, San Jose, CA, USA) with a guard column (5 × 2.0 mm, 5 μm, Thermo Fisher Scientific, San Jose, CA, USA). Isocratic elution was employed for separation. The mobile phase was a mixture of 95.9% deionized water, 3% ACN, and 0.1% TFA pumped with isocratic mode with 0.25 mL/min flow rate. The run time was 8.0 min per sample.

### 3.6. Detection and Quantification

#### 3.6.1. Chromatographic Method

The detection of creatinine was carried out by UV absorbance at 215 nm. Creatinine concentration was calculated from the calibration curve (Section 3.2) using Agilent Chemstation B.01.03 software (Agilent Technology, Santa Clara, CA, United States).

#### 3.6.2. Spectroscopic Method Based on Jaffe Reaction

The absorbance of each sample was measured three times on spectrophotometer (CECIL CE 2501, Cecil Instruments Limited, Cambridge, United Kingdom) at 500 nm after 30 and 90 s. Then, the absorbance difference of samples was compared to the absorbance difference of creatinine standard and creatinine concentration was calculated. For quality control, in each batch, the absorbance of biochemistry control urine was measured.

#### 3.6.3. ELISA Test

The absorbance was measured on the ELISA plate reader (ELx800, BIOTEK Instruments, Bad Friedrichshall, Germany) at 500 nm. Creatinine concentration was calculated from the calibration curve.

### 3.7. Validation

#### 3.7.1. Chromatographic method

The method was validated by evaluating linearity, LOD and LOQ, accuracy (recovery, %), and day-to-day precision (within laboratory reproducibility, RSD, %).

Five-point calibration curves at concentrations of 1, 5, 10, 50, 100 µg/mL were prepared for linearity experiment.

The limit of detection (LOD) and quantification (LOQ) were calculated from the signal-to-noise ratio (S/N) = 3 and S/N = 10, respectively.

The recovery was calculated as a difference between creatinine concentration measured in urine samples and added three levels of known amounts of creatinine standards (10, 20, and 30 µg/mL) into four different urine samples.

Day-to-day precision was evaluated by performing four injections of standard solutions and four different urine samples each day on five different days within two weeks.

To evaluate the potential carry-over, the highest calibrator was injected into the LC–UV instrument, followed by an ACN blank to determine if the results of the solvent blank injections were affected by the previous run.

#### 3.7.2. Spectroscopic Method Based on Jaffe Reaction (Provided by Biosystems)

The detection limit was 0.02 mg/mL creatinine, and the linearity limit was 0.20 mg/mL. Precision is provided by the producer and is given in Table 2.

#### 3.7.3. ELISA Test (Provided by SunLong Biotech Co., Ltd.)

Assay range is 0.2–15.8 mg/mL (2–140 nmol/mL) and LOD 0.06 mg/mL (0.5 nmol/mL). Precision is provided by the producer and is given in Table 3.

### 3.8. Comparison of LC-UV Method with Spectroscopic and ELISA Methods

Twenty-five urine samples (described in Section 3.3) were analyzed with three methods: developed HPLC-UV, spectroscopic method (based on Jaffe reaction), and ELISA test. Each sample was analyzed in three replicates. Additionally, in each batch, the absorbance of biochemistry control urine was measured.

### 3.9. LC-MS/MS Method for Mycotoxin Biomarkers Analysis

The LC-MS/MS method following liquid-liquid extraction was developed and applied for the sensitive determination of eleven mycotoxins (biomarkers of exposure) including zearalenone (ZEN), zearalanon (ZAN), ochratoxin A (OTA), aflatoxins (aflatoxin B1 (AFB1), aflatoxin B2 (AFB2), aflatoxin G1 (AFG1) and aflatoxin M1 (AFM1)), T-2 toxin (T-2), HT-2 toxin (HT-2), sterigmatocystin (STC) and enniatin B (ENB) in pig urine. For mycotoxin quantification internal standard (IS) correction was used.

The method was successfully validated regarding the guidelines specified by the EMEA 2011 in terms of linearity, selectivity, sensitivity (LOD and LOQ), accuracy, precision (intra- and inter-day variability), matrix effect and carry over (publication in preparation).

### 3.10. Standardization of Urine Samples to the Constant Creatinine Level for LC-MS/MS Analysis of Mycotoxin Biomarkers

For each analyte for normalized and not diluted urine, the matrix factor (MF) was calculated for each lot of samples by calculating the ratio of the peak area in the presence of matrix (measured by analyzing blank matrix spiked after extraction with analyte), to the peak area in absence of matrix. The IS-normalized MF was calculated by dividing the MF of the analyte by the MF of the IS. The RSD of the IS-normalized MF calculated from the six lots of samples should be lower than 20% to meet EMEA criteria in terms of matrix effect.

## 4. Conclusions

The described HPLC-UV method was proven to be a rapid and accurate method for the determination of creatinine in pig urine samples. Chromatographic conditions were developed to achieve satisfactory retention of creatinine. Sample preparation based on dilute and shoot approach—the described method is simple and robust.

The developed method has been successfully applied to quantify creatinine in urine samples from 166 pigs. It was also compared with the spectroscopic method and ELISA test. ELISA test couldn’t be reproducible, and the spectroscopic method resulted in 61.5% of the samples in different results as LC-UV method.

Additionally, this developed LC-UV method was successfully applied as a normalization tool in LC-MS/MS mycotoxin biomarkers analysis. The standardization to constant and low creatinine level (0.5 mg/mL) enabled us to achieve similar matrix effects for pig urine samples with different creatinine levels for eleven mycotoxins. This approach seems to be necessary in the case of pig urine because of the high diversity of creatinine level and matrix effects. For human urine, the creatinine levels are rather constant, there could be some exceptions like high creatinine levels (3.4 ± 2.4 mg/mL) for rural subsistence farmers in the former Transkei, South Africa [32]. To sum up, to avoid incorrect mycotoxin biomarker quantification, creatinine levels should be measured with a reliable method in every urine sample.

## Figures and Tables

**Table 1:** The recovery (calculated as a difference between added creatinine standard and creatinine concentration in urine) and day-to-day precision (four injections per sample, each day on five different days within two weeks) of the HPLC method.

Urine Number	Creatinine Concentration [mg/mL]	Recovery [%]			Day-to-Day Precision
		Creatinine Concentration [mg/mL]			RSD [%]
		+0.75 mg/mL	+1 mg/mL	+2 mg/mL	
1	2.49 ± 0.11	91.6	98.5	104	11.49 7.39 3.48 8.28
2	1.56 ± 0.06	93.2	101	108	
3	2.57 ± 0.07	89.4	102	103	
4	1.16 ± 0.10	114	108	111	

**Table 2:** Within run and run to run precision.

	Mean Concentration [mg/mL]	RSD [%]	n
Within Run	0.017	2.9	20
	0.053	1.3	20
Run to Run	0.017	3.9	25
	0.053	2.9	25

**Table 3:** Intra-assay and inter-assay precision.

	Creatinine Concentration	RSD [%]	n
Within Run	Low	<10	20
	Middle		
	High		
Run to Run	Low	<12	8 on 3 different plates
	Middle		
	High		

## Supplementary Materials

The following are available online at https://www.mdpi.com/1420-3049/25/10/2445/s1, Table S1: Human biomonitoring studies, in which the ratio between mycotoxin concentration and creatinine content of urine was calculated.

## References

[1] Wyss M., Kaddurah-Daouk R. (2000). Creatine and creatinine metabolism. Physiol. Rev..

[2] Schwartz G.J., Furth S.L. (2007). Glomerular filtration rate measurement and estimation in chronic kidney disease. Pediatr. Nephrol..

[3] Greenblatt D.J., Ransil B.J., Harmatz J.S., Smith T.W., Duhme D.W., Koch-Weser J. (1976). Variability of 24-Hour Urinary Creatinine Excretion by Normal Subjects. J. Clin. Pharmacol..

[4] Blaszkewicz M., Liesenhoff-Henze K. (2012). Creatinine in urine. MAK-Collection Occup. Heal. Saf. Annu. Threshold. Classif. Work..

[5] Arndt T. (2009). Urine-creatinine concentration as a marker of urine dilution: Reflections using a cohort of 45,000 samples. Forensic Sci. Int..

[6] Liotta E., Gottardo R., Bonizzato L., Pascali J.P., Bertaso A., Tagliaro F. (2009). Rapid and direct determination of creatinine in urine using capillary zone electrophoresis. Clin. Chim. Acta.

[7] Heavner D.L., Morgan W.T., Sears S.B., Richardson J.D., Byrd G.D., Ogden M.W. (2006). Effect of creatinine and specific gravity normalization techniques on xenobiotic biomarkers in smokers’ spot and 24-h urines. J. Pharm. Biomed. Anal..

[8] Katie M. (2017). O’Brien, Kristen Upson, J.P.B. Lipid and Creatinine Adjustment to Evaluate Health Effects of Environmental Exposures. Curr. Env. Heal. Rep..

[9] Suwazono Y., Åkesson A., Alfvén T., Järup L., Vahter M. (2005). Creatinine versus specific gravity-adjusted urinary cadmium concentrations. Biomarkers.

[10] Yeh H.C., Lin Y.S., Kuo C.C., Weidemann D., Weaver V., Fadrowski J., Neu A., Navas-Acien A. (2015). Urine osmolality in the US population: Implications for environmental biomonitoring. Environ. Res..

[11] Boeniger M.F., Lowry L.K., Rosenberg J. (1993). Interpretation of urine results used to assess chemical exposure with emphasis on creatinine adjustments: A review. Am. Ind. Hyg. Assoc. J..

[12] Alessio L., Berlin A., Dell’Orto A., Toffoletto F., Ghezzi I. (1985). Reliability of urinary creatinine as a parameter used to adjust values of urinary biological indicators. Int. Arch. Occup. Environ. Health.

[13] Weaver V.M., Kotchmar D.J., Fadrowski J.J., Silbergeld E., Silbergeld E. (2015). Challenges for environmental epidemiology research: Are biomarker concentrations altered by kidney function or urine concentration adjustment?. J. Expo. Sci. Environ. Epidemiol. Adv..

[14] Jain R.B. (2016). An improved approach to report creatinine-corrected analyte concentrations in urine. Cogent Environ. Sci..

[15] Butler A.R. (1976). The Jaffè reaction: Identification of the colored species. Clin. Chim. Acta..

[16] Lo S.C., Tsai K.S. (1994). Glucose interference in Jaffe creatinine method: Effect of calcium from peritoneal dialysate. Clin. Chem..

[17] Guder W.G., Hoffmann G.E., Hubbuch A., Poppe W.A., Siedel J., Price C.P. (1996). Multicentre evaluation of an enzymatic method for creatinine determination using a sensitive colour reagent. J. Clin. Chem. Clin. Biochem..

[18] Weber J.A., van Zanten A.P. (1991). Interferences in current methods for measurements of creatinine. Clin. Chem..

[19] Mohabbati-Kalejahi E., Azimirad V., Bahrami M., Ganbari A. (2012). A review on creatinine measurement techniques. Talanta.

[20] Miura C., Funaya N., Matsunaga H., Haginaka J. (2013). Monodisperse, molecularly imprinted polymers for creatinine by modified precipitation polymerization and their applications to creatinine assays for human serum and urine. J. Pharm. Biomed. Anal..

[21] Lad U., Khokhar S., Kale G.M. (2008). Electrochemical creatinine biosensors. Anal. Chem..

[22] Fernandes A.R., De Souza P.S., De Oliveira A.E., Chaves A.R. (2018). A new method for the determination of creatinine in urine samples based on disposable pipette extraction. J. Braz. Chem. Soc..

[23] Hušková R., Chrastina P., Adam T., Schneiderka P. (2004). Determination of creatinine in urine by tandem mass spectrometry. Clin. Chim. Acta.

[24] Park E.-K., Watanabe T., Gee S.J., Schenker M.B., Hammock B.D. (2008). Creatinine Measurements in 24 h Urine by Liquid Chromatography-Tandem Mass Spectrometry. J. Agric. Food Chem..

[25] Zhou S., Zuo R., Zhu Z., Wu D., Vasa K., Deng Y., Zuo Y. (2013). An eco-friendly hydrophilic interaction HPLC method for the determination of renal function biomarkers, creatinine and uric acid, in human fluids. Anal. Methods.

[26] Ares A.M., Bernal J. (2012). Hydrophilic interaction chromatography in drug analysis. Cent. Eur. J. Chem..

[27] Thanner S., Czeglédi L., Schwartz-Zimmermann H.E., Berthiller F., Gutzwiller A. (2016). Urinary deoxynivalenol (DON) and zearalenone (ZEA) as biomarkers of DON and ZEA exposure of pigs. Mycotoxin Res..

[28] Binder S.B., Schwartz-Zimmermann H.E., Varga E., Bichl G., Michlmayr H., Adam G., Berthiller F. (2017). Metabolism of zearalenone and its major modified forms in pigs. Toxins.

[29] Gambacorta L., Olsen M., Solfrizzo M. (2019). Pig urinary concentration of mycotoxins and metabolites reflects regional differences, mycotoxin intake and feed contaminations. Toxins.

[30] Ali N., Blaszkewicz M., Degen G.H. (2015). Occurrence of the mycotoxin citrinin and its metabolite dihydrocitrinone in urines of German adults. Arch. Toxicol..

[31] Franco L.T., Petta T., Rottinghaus G.E., Bordin K., Gomes G.A., Alvito P., Assunção R., Oliveira C.A.F. (2019). Assessment of mycotoxin exposure and risk characterization using occurrence data in foods and urinary biomarkers in Brazil. Food Chem. Toxicol..

[32] Shephard G.S., Burger H.-M., Gambacorta L., Gong Y.Y., Krska R., Rheeder J.P., Solfrizzo M., Srey C., Sulyok M., Visconti A. (2013). Multiple mycotoxin exposure determined by urinary biomarkers in rural subsistence farmers in the former Transkei, South Africa. Food Chem. Toxicol..

[33] Njumbe Ediage E., Diana Di Mavungu J., Song S., Wu A., Van Peteghem C., De Saeger S. (2012). A direct assessment of mycotoxin biomarkers in human urine samples by liquid chromatography tandem mass spectrometry. Anal. Chim. Acta.

[34] Ali N., Muñoz K., Degen G.H. (2017). Ochratoxin A and its metabolites in urines of German adults—An assessment of variables in biomarker analysis. Toxicol. Lett..

[35] Rodríguez-Carrasco Y., Mañes J., Berrada H., Font G. (2015). Preliminary estimation of deoxynivalenol excretion through a 24 h pilot study. Toxins.

[36] Ali N., Degen G.H. (2018). Urinary biomarkers of exposure to the mycoestrogen zearalenone and its modified forms in German adults. Arch. Toxicol..

[37] Warth B., Sulyok M., Fruhmann P., Berthiller F., Schuhmacher R., Hametner C., Adam G., Fröhlich J., Krska R. (2012). Assessment of human deoxynivalenol exposure using an LC-MS/MS based biomarker method. Toxicol. Lett..

[38] Warth B., Sulyok M., Berthiller F., Schuhmacher R., Krska R. (2013). New insights into the human metabolism of the Fusarium mycotoxins deoxynivalenol and zearalenone. Toxicol. Lett..

[39] Abia W.A., Warth B., Sulyok M., Krska R., Tchana A., Njobeh P.B., Turner P.C., Kouanfack C., Eyongetah M., Dutton M. (2013). Bio-monitoring of mycotoxin exposure in Cameroon using a urinary multi-biomarker approach. Food Chem. Toxicol..

[40] Warth B., Petchkongkaew A., Sulyok M., Krska R. (2014). Utilising an LC-MS/MS-based multi-biomarker approach to assess mycotoxin exposure in the Bangkok metropolitan area and surrounding provinces. Food Addit. Contam. Part A Chem. Anal. Control. Expo. Risk Assess..

[41] Wallin S., Gambacorta L., Kotova N., Warensjö Lemming E., Nälsén C., Solfrizzo M., Olsen M. (2015). Biomonitoring of concurrent mycotoxin exposure among adults in Sweden through urinary multi-biomarker analysis. Food Chem. Toxicol..

[42] Kwon W., Kim J.Y., Suh S.I., In M.K. (2012). Simultaneous determination of creatinine and uric acid in urine by liquid chromatography-tandem mass spectrometry with polarity switching electrospray ionization. Forensic Sci. Int..

[43] Faulkner W. (2012). Analysis of Creatine and Creatinine on a Porous Graphitic Carbon Column by HPLC/UV. Thermo Fish. Sci. Appl. Note.

[44] Zöllner P., Mayer-Helm B. (2006). Trace mycotoxin analysis in complex biological and food matrices by liquid chromatography–atmospheric pressure ionisation mass spectrometry. J. Chromatogr. A J. Chromatogr. A.

[45] Pinho A.R., Fortuna A., Falcão A., Santos A.C., Seiça R., Estevens C., Veiga F., Ribeiro A.J. (2019). Comparison of ELISA and HPLC-MS methods for the determination of exenatide in biological and biotechnology-based formulation matrices. J. Pharm. Anal..

[46] Barr D.B., Wilder L.C., Caudill S.P., Gonzalez A.J., Needham L.L., Pirkle J.L. (2005). Urinary creatinine concentrations in the U.S. population: Implications for urinary biologic monitoring measurements. Environ. Health Perspect..

[47] Crocker H., Shephard M.D.S., White G.H. (1988). Evaluation of an enzymatic method for determining creatinine in plasma. J. Clin. Pathol..

[48] Jassam N., Weykamp C., Thomas A., Secchiero S., Sciacovelli L., Plebani M., Thelen M., Cobbaert C., Perich C., Ricós C. (2017). Post-standardization of routine creatinine assays: Are they suitable for clinical applications. Ann. Clin. Biochem..

[49] Gutzwiller A., Gafner J.L., Silacci P. (2014). Urinary zearalenone measured with ELISA as a biomarker of zearalenone exposure in pigs. Mycotoxin Res..

[50] Winkler J., Kersten S., Valenta H., Hüther L., Meyer U., Engelhardt U., Dänicke S. (2015). Simultaneous determination of zearalenone, deoxynivalenol and their metabolites in bovine urine as biomarkers of exposure. World Mycotoxin J..

[51] Sunlongbiotech. https://www.sunlongbiotech.com/images/upload/File/PDFNEW/SL0182Po.pdf.

