# Supplementary material for: Dilute-and-Shoot HPLC-UV Method for Determination of Urinary Creatinine as a Normalization Tool in Mycotoxin Biomonitoring in Pigs
Source: Molecules. 2020 May 24;25(10):2445. doi: 10.3390/molecules25102445 (PMC7288144; doi:10.3390/molecules25102445)
Supplement: Supplementary file 1 [file molecules-25-02445-s001.pdf]

Supplementary Material

Table S1. Human biomonitoring studies, in which the ratio between mycotoxin concentration and creatinine content of urine was calculated.

Biomarker	Mean concentration of positive samples [ng mL^{-1}]	Creatinine adjusted concentration [ng mg^{-1} creatinine]	Mean creatinine concentration [mg mL^{-1}]	Creatinine analysis	Matrix effect	Reference
**CIT**	0.03 ± 0.02	0.34 ± 0.36	1.3 ± 0.9	modified Jaffe method	-	[30]
**DH-CIT**	0.10 ± 0.10	1.02 ± 1.10				
**OTA**	0.37 ± 0.28	0.16 ± 0.11	1.7 ± 0.4	Jaffe's reaction	+	[33]
**OTα**	9.03 ± 5.25	4.30 ± 1.75				
**ZEN**	8.45 ± 5.87	7.00 ± 5.37				
**β-ZEL**	13.30 ± 9.16	9.45 ± 8.25				
**DON**	43.70 ± 28.39	29.62 ± 26.40				
**CIT**	6.80	4.50				
**ZEN**	0.529 ± 1.60	0.204 ± 0.456	3.4 ± 2.4	Jaffe's reaction	-	[32]
**FB1**	1.52 ± 2.17	0.185 ± 0.236				
**α-ZEL**	0.614 ± 1.91	0.247 ± 0.590				
**DON**	11.3 ± 27.1	4.94 ± 7.60				
**β-ZEL**	0.702 ± 2.95	0.244 ± 0.820				
**OTA**	0.041 ± 0.086	0.024 ± 0.058				
**DON**	5.38 ± 12.3	7.02 ± 24.4	0.8 ± 0.3	Test™ Creatinine Enzymatic	-	[41]
**DOM-1**	2.32 ± 2.68	1.56 ± 1.26				
**ZEN**	0.09 ± 0.07	0.14 ± 0.13				
**α-ZEL**	0.13 ± 0.26	0.19 ± 0.26				
**β-ZEL**	0.10 ± 0.19	0.13 ± 0.18				
**FB1**	0.07 ± 0.06	0.13 ± 0.26				
**FB2**	0.06 ± 0.12	0.16 ± 0.51				
**OTA**	0.90 ± 0.50	1.43 ± 1.74				
**NIV**	0.13 ± 0.14	0.12 ± 0.07				
**AFM1**	0.05	0.04	1.2 ± 0.3	LC-MS/MS	-	[39]
**FB1**	0.63	0.33				
**OTA**	0.08	0.07				
**DON-15-GlcA**	5.49	5.72				
**DON-3-GlcA**	3.93	3.88				
**ZEN**	0.22	0.21				
**ZEN-14-GlcA**	0.81	0.765				
**NIV**	2.61	2.87				
**OTA**	0.21 ± 0.31	0.17 ± 0.22	1.3 ± 0.1	modified Jaffe method	-	[34]
**OTα**	1.33 ± 2.63	1.01 ± 1.94				
**DON**	18.8 ± 3.5	17.5 ± 2.7	1.1	Jaffe's reaction	-	[35]
**DON-3-GlcA**	4.3	2.4	1.5 ± 0.4	LC-MS/MS	-	[40]
**DON-15-GlcA**	8.1	4.8				
**AFM1**	0.33	0.31				

**ZEN**	0.10 ± 0.05	0.09 ± 0.05		modified		
**α-ZEL**	0.16 ± 0.07	0.16 ± 0.13	1.4 ± 0.9	Jaffe	-	[36]
**β-ZEL**	0.05 ± 0.04	0.04 ± 0.05		method		
